# Cardiovascular Complications of COVID-19: A Scoping Review of Evidence

**DOI:** 10.7759/cureus.48275

**Published:** 2023-11-04

**Authors:** Armand Ntchana, Sanjay Shrestha, Micah Pippin

**Affiliations:** 1 Family Medicine-Alexandria, Louisiana State University Health Sciences Center, Alexandria, USA

**Keywords:** long-term effects of covid-19, acute effects of covid-19, cardiovascular complications, cardiac complications, covid-19

## Abstract

This scoping review sought to identify the nature and extent of clinical evidence regarding the acute and long-term cardiovascular complications associated with COVID-19. Forty-nine studies published between 2020 and 2023 were selected for review. The studies were divided into two groups. The referential group included 22 studies. The second group of 27 studies was used for a detailed review to assess the strength of the evidence. The aggregate evidence indicates that the most common cardiac complications associated with COVID-19 include but are not limited to acute pericarditis, acute myocardial injury, acute myocarditis, various arrhythmias, microvascular angiopathy, left ventricular dysfunction, heart failure, acute cardiac injury, and acute coronary syndrome. Clinical and epidemiological implications of the findings are investigated, and future research recommendations are proposed.

## Introduction and background

Since December 2019, the world has been facing a new pandemic, the severe acute respiratory syndrome coronavirus 2 (SARS‐CoV‐2). The 2019 coronavirus disease, eventually named COVID-19, first appeared in Wuhan, China [1-3]. The WHO officially declared COVID-19 a pandemic in March 2020 [4-5]. On May 5, 2023, more than three years after COVID-19 was designated as a pandemic, the global public health emergency was declared over by the WHO. Globally, as of July of 2023, there have been over 768 million clinically confirmed cases of COVID-19, which resulted in almost 7 million deaths [6]. All 50 states and territories have been affected [7]. However, the prevalence and incidence rates vary significantly across states [8-10]. To date, there have been 103.4 million clinically confirmed cases of COVID-19 in the United States and 1.14 million deaths directly attributable to COVID-19 [7].

According to its phylogenetic tree, SARS-CoV-2 is a member of the same coronavirus family as Middle East Respiratory Syndrome -Corona Virus (MERS-CoV) and the severe acute respiratory syndrome coronavirus (SARS-CoV) [1-2]. Despite the fact that the majority of COVID-19 clinical manifestations have been respiratory [11-13], as the number of infected patients increased, there have been several clinical reports of significant cardiac problems in a sizable percentage of COVID-19 patients [14-18]. According to mounting clinical data, SARS-CoV2 infection is thought to be linked to a number of proinflammatory mediators that may be crucial in the pathogenesis of cardiac problems [19-21].

Two causative mechanisms of cardiac complications associated with SARS-COV-2 were proposed. Precisely, it was initially speculated that cardiac complications in a sizable minority of patients with COVID-19 could be caused by direct cardiomyocyte infection, eventually resulting in cardiomyocyte death [22-24]. However, subsequent clinical and epidemiological data analyses did not support this causative mechanism [14,25]. Alternatively, others hypothesized that indirect mechanisms such as ischemia, fever, adrenergic hyperactivity, or inflammation secondary to cytokine storm and COVID-19 hyperinflammatory syndrome might mediate cardiac injury in patients with COVID-19 [12,26-30]. Although the clinical evidence has grown, especially in the last two years, three important questions remain unresolved and require further research and analysis. The first question pertains to the exact causes of cardiac complications in patients with COVID-19. It also remains unclear whether the same causative factors are responsible for cardiac complications for all populations of COVID-19 patients. 

In addition to the unsettled questions of the etiology of cardiac complications in patients with COVID-19, the issues of the extent, severity, and long-term effects of cardiac complications in this cohort of patients still need to be fully understood and require urgent investigation. It also needs to be stated that much of the current knowledge of SARS-CoV-2 and its effects on the cardiovascular system is derived from the epidemics that predated the COVID-19 pandemic, and specifically from SARS-CoV, MERS-CoV, and H1N1 influenza. During these epidemics, there were reports of a significant association between underlying cardiovascular disease, myocardial injury, and adverse patient outcomes [17,21,31].

Given the ongoing urgency of the topic and the need to map out the growing but complex and heterogeneous evidence from recent studies, this scoping review aims to provide a focused overview of cardiovascular complications associated with COVID-19, heart failure, myocardial injury, myocarditis, acute myocardial infarction, dysrhythmias, and venous thromboembolic events. 

## Review

Search strategy

A wide-ranging search for articles published in English in peer-reviewed medical journals using PubMed, EMBASE, and Cochrane Library repositories and utilizing the keywords "COVID-19", "SARS-CoV-2", "heart," "cardiac," "cardiovascular," "complications," "myocarditis," "acute myocardial infarction," "acute coronary syndrome," "dysrhythmia," "arrhythmia," "heart failure," "venous thromboembolism," was conducted. In total, 49 sources published between 2020 and 2023 were selected, with 11 studies (22.4%) published in 2020, 10 studies (20.4%) published in 2021, 19 studies (38.8%) published in 2022, nine studies (18.4%) appeared in 2023. The studies in the scoping review were divided into two groups. The referential group included 22 studies used to gauge the size and scope of available clinical literature on the topic. The second group of studies was used for a detailed review to assess the strength of the evidence. The detailed review was conducted on 27 studies, which included seven case series, which accounted for 25.9% of sources in the detailed review, six meta-analyses (22.2%), five systematic reviews (18.5%), five comprehensive reviews (18.5%), two scoping reviews (7.4%), one narrative review (3.7%), and three studies or 14.3% were both systematic reviews and meta-analyses. All 49 studies in the scoping review focused explicitly on adverse cardiovascular effects and acute and long-term complications associated with COVID-19. 

Cardiac complications in patients with COVID-19

The cumulative evidence gathered in the current review suggested that the relationship between COVID-19 and the cardiovascular system appears highly complex and, to date, not completely understood, despite constantly growing clinical and epidemiological evidence.

However, unlike at the start of the pandemic in 2020, the aggregate evidence indicates that the most common cardiac complications associated with COVID-19 include but not limited to acute pericarditis [19,32], acute myocardial injury [14-15,19-20], acute myocarditis [28,33-34] various arrhythmias [28,34-36], microvascular angiopathy [19,37], left ventricular dysfunction [15,19], heart failure [16-17,33,36,38-39], acute cardiac injury [15,28,38,40], acute coronary syndrome [20,34,40]. The detailed review is presented in Table 1.

**Table 1:** Summary of Findings on Cardiac Complications Associated with COVID-19 [1,14-21,25,28,30,33,35-40,44-50]

Authors	Design	Main Findings
Aghagoli et al., [19]	Case series N = 13	COVID‐19 patients with pre-existing cardiovascular disease are counted in greater frequency in ICUs and suffer higher mortality rates. Cardiac presentations for COVID‐19 include acute pericarditis, left ventricular dysfunction, and acute myocardial injury.
Alqahtani et al., [37]	Case series N = 117	COVID-19 is positively associated with heart failure, arrhythmias, microvascular angiopathy, and long-term cardiac damage.
Ammirati et al., [14]	Case series N = 23	Acute myocarditis (AM) is a rate cardiovascular complication of COVID-19. AM prevalence among hospitalized COVID-19 patients was 2.4/1000 hospitalizations (definite) and 4.1/1000 (possible). The median age of definite cases was 38 years, and 38.9% were female. Thirty-one cases (57.4%) occurred in the absence of COVID-19-associated pneumonia. The composite of in-hospital mortality or temporary mechanical circulatory support occurred in 20.4%. At 120 days, estimated mortality = 6.6%.
Ashton et al., [46]	Systematic Review N = 9	Existing data indicate an increased risk of severe complications and mortality in those who contract COVID-19 with pre-existing CV disease (CVD) or who present with risk factors such as hypertension, diabetes mellitus, hypercholesterolemia, and obesity. A significant risk factor for respiratory failure necessitating mechanical ventilation was also determined to be the high prevalence of obesity among COVID-19 hospital patients. It became clear that acute cardiac injury (myocarditis, pericarditis, and reduced ventricular function) following infection with COVID-19 was frequent as the evidence and incidence of the multisystem nature of COVID-19 became known. Long-term cardiac symptoms like dyspnea and tiredness (36%), palpitations (20%), and unusual chest discomfort (17%) are all linked to COVID-19. Due to its association with high morbidity and the worsening of underlying CV problems, Long COVID is concerning for patients who have CVD.
Brogi et al., [15]	Systematic Review N = 49 Acute cardiac injury (ACI) (n = 20) Heart failure (n = 10) Myocardial infarction (n = 7) Takotsubo syndrome (n = 6) Myocarditis (n = 12) Pericardial effusion (n = 5) Arrhythmias (n = 13) Right ventricular dysfunction (n = 6) Meta-Analysis N = 7 The RR = 0.20. 95% CI: [0.17; 0.24], p < 0.00001, I^2^ = 0.75.	The most common cardiac problem was ACI (20–45%). Acute cardiac injury (ACI) is an independent risk factor for severe forms of SARS-CoV-2 infection and an independent predictor of mortality. Possible arrhythmic alterations (incidence 3-60%) have to be taken into account for the potential complications and the ensuing hemodynamic instabilities. Patients with acute cardiac injury are significantly older, have more comorbidities, are more likely to develop complications, and have higher mortality rates. The most prevalent comorbidity is hypertension (30-59.8%). This group of individuals had a high rate of CVD (up to 57%) and only 10% had coronary artery disease. Patients with CVD showed greater rates of ICU admission, severe form prevalence, and mortality.
Cannata et al., [25]	Meta-Analysis N = 15 The RR = 0.62. 95% CI: [1.20; 2.20], p = 0.002.	Mortality among patients with CV diseases was higher relative to periods outside the pandemic, independent of co-infection with COVID-19. The effect was larger in studies with the biggest decline in admission rates, suggesting a sicker cohort of patients in this period.
Chang et al., [28]	Comprehensive Review (N = 35) Cardiomyopathy (n = 1) Myocarditis (n = 6) Heart failure (n = 1) Arrhythmia (n = 1) Thromboembolism (n = 2) Cardiac events (n = 24)	SARS-CoV-2 may either induce new cardiac pathologies and/or exacerbate underlying cardiovascular diseases as the high inflammatory burden of COVID-19. The presence of cardiac injury, heart failure, and myocarditis are independent factors associated with mortality.
Denegri et al., [35]	Case series N = 902	Arrhythmias (AF) are quite common at admission in COVID-19 infection and have been associated with worse prognosis. AF at baseline ECG determined a significantly higher risk of mortality compared to other rhythms and even to the history of AF.
Jafari-Oori et al., [16]	Meta-Analysis N = 26	Patients with cardiac issues with COVID-19 are significantly more likely than those without to get severe disease, require ICU admission, or pass away. Patients with cardiac injury who have COVID-19 are more likely to develop a severe form of the disease, be admitted to the intensive care unit, or pass away. The pooled rates for AMI were 21%, heart failure was 14%, arrhythmia was 16%, cardiac arrest was 3.46%, and acute coronary syndrome was 1.3% in COVID-19. AMI and shock had a combined incidence of 33% in individuals with severe illness.
Kochi et al., [1]	Case Series N = 6	Arrhythmic problems and myocardial damage are both brought on by COVID-19 infection.
Kole et al., [47]	Comprehensive Review N = 25	Clinical manifestations of COVID-19 during acute and post-acute syndrome include myocardial injury, dysrhythmias, atrial arrhythmia, AV block, sinus tachycardia, sinus bradycardia, heart failure, coagulation abnormalities (DVT, PE, VTE). Other complications include pericarditis, pericardial effusion, and Takotsubo syndrome. Poor research has been done on post-COVID-19 syndrome, which affects COVID-19 survivors of various severities of disease and ages. Chest tightness, cardiac arrhythmias, palpitations, hypotension, an elevated heart rate, venous thromboembolic disorders, myocarditis, and acute/decompensated HF were all associated with cardiovascular events.
Kunutsor et al., [20]	Systematic Review (N = 17) Meta-Analysis (N = 17)	For COVID-19 associated: § Heart failure (n = 4): RR = 17.6%; 14.2-21.2; I^2^ = 32%; 95% CI 0, 76%; p = 0.20. § Myocardial injury (n = 11): RR = 11.8%; I^2^ = 87%; 95% CI 79, 92%; p < 0.01. § Cardiac arrythmia (n = 6): RR = 9.3%; 5.1-14.6; I^2^ = 78%; 95% CI 52, 90%; p < 0.01. § Acute Coronary Syndrome (n = 2): RR = 6.2% (1.8-12.3). The incidence of myocardial injury was higher in older age groups and groups with a higher prevalence of pre-existing hypertension; the incidence of myocardial injury was similar in groups with a high or low prevalence of pre-existing CVD.
Long et al., [17]	Case series N = 37	COVID-19 is associated with myocardial injury and myocarditis, AMI, heart failure, arrhythmias, and VTE.
Nadarajah et al., [48]	Meta-Analysis (N = 158)	The collateral damage of COVID-19 to CV clinical services has been high. There have been fewer procedures, hospitalizations, procedures, and consultations and increased mortality among patients with CV conditions.
Osoro et al., [49]	Scoping Review N = 44	Cardiovascular complications noted in COVID-19 patients include myocardial infarction, myocarditis, arrhythmia, myocardial interstitial fibrosis, endothelial cell dysfunction, vasculitis, thromboembolism, and dysautonomia. Overall, the development of cardiovascular complications in COVID-19 patients worsened the pandemic situation.
Peiris et al., [40]	Scoping Review N = 63	The overall frequency of acute cardiac injury ranged from 15% to 33%. The main cardiac complications were arrhythmias (3.1% - 6.9% in non-severe patients, 33.0% - 48.0% in severe disease), acute coronary syndromes (6% - 33% in severe disease, and myocarditis.
Ramadan et al., [33]	Case Series N = 73	COVID-19 is positively associated with higher rates of cardiac complications (CC). Most common CC: myocarditis, heart failure, myocardial injury, arrhythmia. Patients with a prior history of CVD appear to be more susceptible to CC.
Saha et al., [50]	Contemporary Review N = 68	A variety of arrhythmic manifestations in patients with COVID-19 range from relatively benign conditions such as transient sinus bradycardia to potentially life-threatening conditions such as ventricular arrhythmias and sudden cardiac death. Atrial fibrillation is the most common arrhythmia seen in acute COVID-19 patients.
Sahranavard et al., [36]	Systematic Review N = 22 Meta-Analysis N = 22	COVID-19 is associated with a higher incidence of various cardiac complications: Arrhythmia (n = 3): RR = 10.11; CI 95% [5.12; 19.00], p < 0.001, I^2^ = 75.21. Heart Failure (n = 4); RR = 22.34; CI 95% [14.05; 33.60], p < 0.001, I^2^ = 79.44. Myocardial Injury (n = 13); RR = 17.85; CI 95% [13.18; 23.72], p < 0.001, I^2^ = 86.84.
Salabei et al. [32]	Case series N = 89	The cardiac complications of COVID-19 most frequently include myopericarditis leading to shock and increased morbidity and mortality.
Shafi et al., [21]	Systematic Review N = 61	Evidence supports a clear correlation between cardiovascular disease and COVID‐19 severity. Hypertension and diabetes are the most prevalent comorbidities associated with adverse cardiovascular outcomes. Cardiac manifestations are an important aspect of disease manifestation in COVID‐19, with atrial fibrillation, myocarditis, heart failure, and cardiogenic shock the most commonly reported manifestations.
Sousa Rêgo et al., [44]	Narrative Review N = 34	The most frequent cardiovascular complication was acute cardiac damage, which was documented in 28 (25.9%) patients. Heart failure, cardiogenic shock, and acute coronary syndrome were each reported in 4 (2.8%) patients, while pericardial effusion was observed in 2 (1.9%). Stress cardiomyopathy may be a serious consequence of COVID-19 due to its association with a systemic cytokine storm. Late cardiovascular complications include myocardial inflammation, regional scar, and pericardial enhancement.
Srinivasan et al., [34]	Case Series N = 83	Although the incidence of cardiac complications in non-comorbid patients with COVID-19 may be considered low, its significance should not be underestimated when considering the potential impact on healthcare systems. Most common COVID-19-associated cardiac complications: § Direct complications such as direct cardiotoxicity, microvascular dysfunction, acute coronary syndrome, heart failure, myocarditis, arrhythmia, and venous thromboembolism. § Indirect complications such as long-term inflammatory changes, hypoxemia-mediated effects, and supply-demand mismatch.
Toloui et al., [38]	Systematic Review N = 40 Meta-Analysis N = 40	Acute cardiac injury, heart failure, and cardiac arrest were all more common than they were in the general population: 19.46% (95% CI: 18.23-20.72), 19.07% (95% CI: 15.38-23.04), and 3.44% (95% CI: 3.08-3.82), respectively. When patients have acute cardiac damage, the overall odds of morality RR is 14.24 (95% CI: 8.67-23.38). When the study was restricted to those with aberrant serum troponin levels, the pooled odds ratio for mortality was 19.03 (95% CI: 11.85-30.56). In COVID-19 patients, acute cardiac damage and elevated serum troponin levels were the most frequent cardiac consequences.
Vosko et al., [30]	Comprehensive Review	Cardiovascular sequelae of COVID-19 and long-term CVD risk modification include myocarditis, acute coronary syndrome, heart failure, thromboembolic complications, and arrhythmia.
Welty et al., [18]	Comprehensive Review N = 68	The relationship between cardiovascular diseases and COVID-19 is bidirectional, which implies that pre-existing cardiovascular comorbidities increase the morbidity and mortality of COVID-19. Newly emerging cardiac injuries occur in the settings of acute COVID-19 in patients with no pre-existing cardiovascular disease.
Zuin et al., [39]	Systematic Review N = 6 Meta-Analysis N = 6	Acute HF represents a frequent complication of COVID-19 infection associated with a higher risk of mortality in the short-term period. The polled incidence of heart failure (HF) as a cardiac complication in COVID-19 patients was 20.2% of cases (95% CI: 11.1–33.9%, p < 0.0001 I^2^ = 94.4%). The related mortality risk in COVID-19 patients OR = 9.36% (95% CI: 4-76-18.4, p < 0.0001, I^2^ = 56.6%. Age was used as a moderator variable in the meta-regression analysis, but it was unable to establish a statistically significant correlation with either the mortality risk among the same participants (p = 0.053) or the incidence of acute HF onset as a consequence of COVID-19 disease (p = 0.062).

It should be noted that the authors of all 27 studies reviewed in detail made three general conclusions regarding cardiac complications in patients with COVID-19: (a) the prevalence of specific cardiovascular complications appears to vary depending on specific gender and some other socioeconomic characteristics of patients but not their age; (b) the rate of cardiac complications is generally statistically significant across the board, regardless of the statistical analysis used; and (c) COVID-19 related cardiac complications are generally associated with increased mortality rates compared to patients with only pulmonary complications. 

Discussion

As detailed earlier, the scoping review of the cumulative evidence suggested that COVID-19 is associated with many acute and long-term cardiovascular complications. However, the nature of the current clinical and observational evidence does not allow for making reliable causative claims. At the same time, the sheer amount of clinical evidence supporting the causative link between COVID-19 and cardiovascular complications has been constantly increasing. Also, from the reliability viewpoint, the research on COVID-19-associated cardiovascular complications has advanced substantially since the pandemic's start. The research has progressed from the appearance of the first case reports linking COVID-19 with various cardiovascular complications across virtually all population groups to the publication of more comprehensive and informative clinical studies. Such developments promise that the causative connection between the SARS-CoV-2 virus and cardiovascular complications will be established, and as a result, better disease management protocols will be developed.

In addition to the link between COVID-19 and cardiovascular complications, the current review also highlighted the need to explore an additional clinical factor in this relationship - probable adverse cardiovascular effects of COVID-19 vaccines. Specifically, as the bulk of the populations in various countries, including the U.S., have received several rounds of vaccinations against COVID-19, multiple studies appeared in the extant medical literature [30,41-45] reporting credible clinical evidence that suggested that the administration of COVID-19 vaccines may be associated with several significant cardiovascular complications across various population groups. At present, given the limitations of clinical data and inconsistencies in data collection, it is unclear whether (a) the observed association is due to the effects of the vaccination only or (b) the available evidence points to compound interactive effects of previously undiagnosed cardiovascular conditions in individuals who had never had COVID-19 and vaccines. It also needs to be established if there is a link between certain types of COVID-19 vaccines and subsequent cardiovascular complications. The number of immunization rounds may be another factor in the relationship, especially for different population groups.

The review also underlined the issue of the prevalence of COVID-19-associated cardiovascular complications in different age groups. The current evidence suggests that if individuals with pre-existing cardiovascular conditions and individuals older than 65 years of age are removed from epidemiological analyses, the prevalence of COVID-19-associated cardiovascular complications appears to be about the same in virtually all remaining age groups, at least based on available data. This observation is odd and needs to be explained, preferably using such analytical techniques as cluster analysis and/or discriminant analysis. In any case, more robust epidemiological models with better operationalized factors are urgently needed. 

Lastly, the initial case reports and reviews of clinical studies of COVID-19 patients focused primarily on pulmonological symptoms and hematopathological manifestations of the disease. The growing clinical evidence suggests that cardiovascular complications appear fast and have equally dire consequences for patients. Therefore, the current review highlighted the need for clinicians to develop and employ practical multi-faceted treatment approaches during the acute stage of COVID-19. Likewise, it is necessary to have more holistic case management of the long-term effects of COVID-19 disease. 

Future directions 

This scoping review has highlighted that although the number of primary studies that explored the cardiovascular effects of COVID-19 has been gradually growing as more clinical evidence became available, the majority of extant studies on the topic are either observational or employed relatively simple case-study designs. Thus, more high-quality clinical research currently needs to examine the acute and long-term cardiovascular implications of COVID-19. As the world emerges from the COVID-19 pandemic, more rigorous studies examining the long-term consequences of COVID-19 are needed. More detailed studies are needed, specifically focusing on the clinical management of acute and long-term cardiac damage due to COVID-19. 

## Conclusions

The severity, extent, and long-term cardiovascular effects of COVID-19 are yet to be fully understood by clinicians. Despite gradually accumulating evidence from clinical and observational studies, research on the acute and long-term cardiovascular implications of COVID-19 remains limited. There is also a need for more focus on the causative factors of cardiac complications associated with both acute and long-term COVID-19 as the academic and medical communities attempt to understand and develop effective treatment approaches. COVID-19 can have damaging severe long-term effects on a person’s quality of life. Overall, the currently available evidence suggests that COVID-19 is associated with a high risk of cardiovascular complications regardless of individual demographic characteristics or cardiovascular morbidities. Additionally, the evidence on long-term COVID-19 shows that practically all patients require ongoing follow-up and treatment even after utilizing a thorough method. Patients with severe COVID-19, those with multisystem inflammatory syndrome during or after COVID-19, those over 65, and those with underlying comorbidities like respiratory disease, obesity, diabetes, hypertension, chronic cardiovascular disease, chronic kidney disease, post-organ transplant, or active cancer should receive priority care, though, as these populations are at high risk for long-term cardiovascular implications.
